# Special Issue “Ion Conductance and Ion Regulation in Human Health and Disease”

**DOI:** 10.3390/ijms262110650

**Published:** 2025-11-01

**Authors:** Lars Kaestner

**Affiliations:** 1 Theoretical Medicine and Biosciences, Medical Faculty, Saarland University, 66424 Homburg, Germany; lars_kaestner@me.com; 2 Dynamics of Fluids, Experimental Physics, Saarland University, 66123 Saarbruecken, Germany

Ion concentration gradients across the cell membrane are fundamental to numerous processes that define life. These gradients drive electrical activity, signal transduction, and transport mechanisms essential for cellular function. Consequently, a deep understanding of ion conductance and regulation is vital for unraveling both physiological and pathophysiological mechanisms at the cellular level. This Special Issue explores the molecular architecture and functional dynamics of ion transport across biological membranes, with particular emphasis on intracellular ion stores and signaling pathways governed by ion fluxes. The scope spans a wide range of cell types—from highly specialized excitable cells such as cardiac myocytes to the virtually organelle-free red blood cells (RBCs). Although these cell types may appear to represent opposite ends of the cellular complexity spectrum, they share a surprising number of functional properties, particularly in their reliance on finely tuned ion channel activity [1]. Ion channels and Ca^2+^ signaling, in particular, emerge as central regulators of cellular physiology [2]. They influence key processes such as immune responses [3], electrical excitability [4] and gene expression [5]. The precise modulation of these channels is not only essential for maintaining cellular homeostasis but also offers critical insights into the mechanisms underlying various diseases. By bridging molecular insights with physiological relevance, this Special Issue aims to highlight the integrative role of ion transport in health and disease, and to inspire future research into its therapeutic potential.

Three contributions in this Special Issue focus on the intermediate-conductance Ca^2+^-activated K^+^ channel encoded by the *KCNN4* gene [6]. At the same time, this underscores the challenges faced by the field of electrophysiology in effectively communicating its findings and concepts: Notably, the three articles refer to the *KCNN4*-encoded channel using three different names: Gárdos channel (contribution 1), SK4 (contribution 2), and K_Ca_3.1 (contribution 3). The Gárdos channel is named after the Gárdos effect—Ca^2+^-induced K^+^ loss in RBCs [7]—which was discovered before the concept of ion channels had even been proposed [8] and well before the Gárdos channel in RBCs was identified as the product of the *KCNN4* gene [9]. SK4 stands for small-conductance Ca^2+^-activated K^+^ channel subtype 4, a term that first appeared in the scientific literature in the early 2000s [10,11]. The transition to the K_Ca_3.1 nomenclature was part of a broader initiative by the International Union of Pharmacology (IUPHAR) to establish a unified naming system for ion channels, aligning terminology with molecular identity and functional characteristics [12]. For the sake of consistency, this Editorial adopts the nomenclature used in each referenced article.

In RBCs, the Gárdos channel and the mechanosensitive ion channel Piezo1 play pivotal roles in regulating cellular deformability and may also contribute actively to thrombus formation [13]. These channels are essential for maintaining ionic homeostasis and membrane potential, which in turn influence the mechanical properties of RBCs as they navigate through the microvasculature [14,15,16] and splenic filtration slits [17]. Using automated patch clamp technology combined with transcriptomic and proteomic profiling, Petkova-Kirova et al. (contribution 1) demonstrated that both channels are functionally active in reticulocytes and mature RBCs, despite their low copy numbers per cell [18]. Interestingly, Gárdos channel activity appears to be more prominent in reticulocytes, suggesting a developmental regulation, whereas Piezo1 activity predominates in mature RBCs, aligning with its role in mechanotransduction during circulation [14,15,16,17]. Although their direct interaction cannot be captured via patch clamp methods, the functional interplay between the Gárdos channel and Piezo1 significantly influences membrane potential and the capacity of RBCs to deform and pass through narrow capillaries or splenic sinusoids [17]. This mechanistic insight is particularly relevant for understanding hematological disorders such as hereditary xerocytosis and sickle cell disease, where altered ion channel activity contributes to impaired RBC survival and increased thrombotic risk [19,20] and [21,22], respectively.

Ca^2+^ sensing is further exemplified by the SK4 channel, which is directly gated by calmodulin (CaM) [23]. Upon binding Ca^2+^, CaM undergoes a conformational change that enables it to activate SK4, thereby linking intracellular Ca^2+^ dynamics to membrane potential regulation [24]. Segura et al. (contribution 2) demonstrated that mutations within Helix B of SK4 disrupt CaM binding, leading to a marked suppression of K^+^ currents. Remarkably, these functional deficits can be rescued by engineered CaM variants, underscoring the specificity and adaptability of CaM-channel interactions. These findings highlight the critical role of electrostatic forces and structural compatibility in Ca^2+^-dependent gating mechanisms. They also emphasize how subtle alterations in channel architecture or regulatory protein structure can profoundly impact ion channel function. Given the involvement of SK4 in immune cell activation, epithelial transport, and cardiovascular regulation, understanding its molecular regulation opens new avenues for therapeutic intervention in diseases where Ca^2+^ signaling is dysregulated. This work not only advances our knowledge of SK4 channel biology but also illustrates the broader principle that ion channel modulation often hinges on finely tuned protein–protein interactions.

In CD8^+^ T-cells, which play a critical role in tumor surveillance and are central to immunotherapeutic strategies, K^+^ channels K_v_1.3 and K_Ca_3.1 are essential for maintaining the negative membrane potential required for Ca^2+^ influx through Ca^2+^ release-activated channels (CRAC) [25]. This Ca^2+^ signaling is vital for T-cell activation, proliferation, and effector function [26,27]. The recent findings by Jusztus et al. (contribution 3) revealed that ovarian cancer patients exhibit elevated expression of K_v_1.3 and diminished activity of K_Ca_3.1 in their CD8^+^ T-cells. This imbalance correlates with exaggerated Ca^2+^ responses, which may reflect dysregulated immune signaling and compromised cytotoxic function. These alterations suggest that K_v_1.3 and K_Ca_3.1 not only serve as functional regulators of T-cell physiology but may also act as diagnostic biomarkers for immune competence in cancer patients. Understanding the molecular mechanisms underlying this channel imbalance could provide valuable insights into immune evasion in tumors and inform the development of targeted therapies aimed at restoring effective T-cell responses. Moreover, modulation of these channels may offer novel avenues for enhancing the efficacy of immunotherapies in oncology.

In cardiac physiology, catecholaminergic polymorphic ventricular tachycardia (CPVT) is a life-threatening arrhythmic disorder associated with gain-of-function mutations in the ryanodine receptor 2 (RyR2), a key regulator of intracellular Ca^2+^ release in cardiac myocytes [28]. These mutations lead to abnormal Ca^2+^ handling and spontaneous Ca^2+^ release events [29], which can trigger ventricular arrhythmias under adrenergic stress. Flecainide, traditionally known as a Na^+^ channel blocker, has emerged as an effective therapeutic agent for CPVT [30,31]. Beyond its action on Na^+^ channels, Gaburjakova et al. (contribution 4) showed that flecainide selectively inhibits RyR2-mediated countercurrents—charge-balancing ionic movements that accompany Ca^2+^ release—without impairing the receptor’s primary channel activity. This nuanced modulation of intracellular ion fluxes may stabilize Ca^2+^ homeostasis and reduce arrhythmogenic potential. These findings suggest that flecainide’s efficacy in CPVT may stem not only from its antiarrhythmic properties at the membrane level but also from its ability to fine-tune intracellular ion dynamics. This dual mechanism highlights the therapeutic value of targeting sub-conductance states and accessory currents in ion channelopathies.

On the genetic front, the *KCNQ2* gene—implicated in a spectrum of epileptic syndromes ranging from benign familial neonatal epilepsy to severe developmental and epileptic encephalopathies —exemplifies the complexity of variant interpretation in clinical genomics [32]. Accurate classification of missense mutations remains a major challenge due to the gene’s functional diversity and the subtlety of pathogenic mechanisms [33]. Saez-Matia et al. (contribution 5) addressed this issue by developing MLe-KCNQ2, a machine learning ensemble model that integrates genomic features with a Variant Frequency Index to predict the pathogenicity of *KCNQ2* missense variants. The model demonstrated high specificity and sensitivity in distinguishing benign from disease-causing mutations, offering a robust tool for variant prioritization in diagnostic workflows. This gene-specific approach represents a significant advancement in precision medicine, where tailored computational models can complement clinical and functional data to improve diagnostic accuracy. Moreover, it underscores the broader need for customized predictive frameworks that account for gene-specific biology, variant context, and population-level data. As genomic sequencing becomes increasingly routine in clinical settings, such tools will be essential for translating genetic information into actionable insights for patient care.

Finally, the multifunctional protein Anoctamin 6 (ANO6) plays dual roles as a phospholipid scramblase and a Ca^2+^-activated ion channel, positioning it as a key regulator of membrane dynamics and intracellular Ca^2+^ signaling [34,35]. ANO6 not only modulates Ca^2+^ flux but also influences the expression and function of other membrane proteins, including the Cystic Fibrosis Transmembrane Conductance Regulator (CFTR) [36], thereby impacting a wide range of physiological processes [37,38]. Studies in HEK293 cells by Ousingsawat et al. (contribution 6) demonstrated that endogenous expression of ANO6 significantly alters the functional behavior of overexpressed anoctamin family members. These findings underscore the critical importance of cellular context in experimental design and interpretation, as the presence of native regulatory proteins can profoundly influence the activity and interactions of introduced constructs. The dual functionality of ANO6 highlights the complexity of ion channel regulation and its integration with lipid remodeling and protein trafficking. Its role in shaping the cellular microenvironment suggests broader implications for epithelial transport, immune responses, and disease mechanisms such as cystic fibrosis and cancer. Future research into ANO6 and its network of interactions may reveal novel therapeutic targets and enhance our understanding of how multifunctional membrane proteins coordinate cellular signaling landscapes.

This Special Issue underscores the remarkable versatility of ion conductance and regulation across a wide spectrum of physiological and pathological contexts. The studies presented not only deepen our understanding of molecular mechanisms but also highlight the translational potential of targeting ion channels for diagnostic and therapeutic purposes (all contributions). Looking ahead, the field of ion transport biology will likely have a prosperous future. Emerging technologies such as automated electrophysiology based on planar chips ([39] and contribution 1), single-cell omics [40], and machine learning-based variant interpretation ([41] and contribution 5) are opening new avenues for dissecting ion channel function with unprecedented precision. Moreover, the interplay between ion channels and cellular context—whether developmental stage, tissue type, or disease state—demands further investigation. Future research should aim to unravel how mechanical forces, metabolic cues, and genetic variation converge on ion transport systems to shape cell behavior and systemic physiology. I hope that this Special Issue inspires continued exploration into the molecular and physiological regulation of ion signaling, fostering interdisciplinary dialog and innovation in biomedicine. The transition from ion gradients to clinical impact is far from complete—and the discoveries yet to come promise to be as electrifying as the ion currents themselves.
